# Glucocorticoid modulatory element-binding protein 1 (GMEB1) interacts with the de-ubiquitinase USP40 to stabilize CFLAR_L_ and inhibit apoptosis in human non-small cell lung cancer cells

**DOI:** 10.1186/s13046-019-1182-3

**Published:** 2019-05-02

**Authors:** Wentao An, Shun Yao, Xiaoyang Sun, Zhaoyuan Hou, Yidan Lin, Ling Su, Xiangguo Liu

**Affiliations:** 1 Shandong Provincial Key Laboratory of Animal Cell and Developmental Biology, School of Life Sciences, Shandong University, Room N8-110, 72 Binhai Road, Qingdao, 266237 People’s Republic of China; 2 Shandong Provincial Collaborative Innovation Center of Cell Biology, School of Life Sciences, Shandong Normal University, Jinan, China; 3 Department of Biochemistry and Molecular Cell Biology, Shanghai Jiaotong University School of Medicine, Shanghai, China; 4 Thoracic Surgery Department of West China Hospital, West China Medical School of Sichuan University, Chengdu, China

**Keywords:** GMEB1, USP40, de-ubiquitination, CFLAR_L_, NSCLC cells, Apoptosis

## Abstract

**Background:**

GMEB1 was originally identified via its interaction with GMEB2, which binds to the promoter region of the tyrosine aminotransferase (*TAT*) gene and modulates transactivation of the glucocorticoid receptor gene. In the cytosol, GMEB1 interacts with and inhibits CASP8, but the molecular mechanism is currently unknown.

**Methods:**

Human non-small cell lung cancer cells and 293FT cells were used to investigate the function of GMEB1/USP40/CFLAR_L_ complex by WB, GST Pull-Down Assay, Immunoprecipitation, Immunofluorescence and Flow cytometry analysis. A549 cells overexpressing green fluorescent protein and GMEB1 shRNA were used for tumor xenograft using female athymic nu/nu 4-week-old mice.

**Results:**

We found GMEB1 interacted with CFLAR_L_ (also known as c-FLIP_L_) in the cytosol and promoted its stability. USP40 targeted CFLAR_L_ for K48-linked de-ubiquitination. GMEB1 promoted the binding of USP40 to CFLAR_L_. *USP40* knockdown did not increase CFLAR_L_ protein level despite GMEB1 overexpression, suggesting GMEB1 promotes CFLAR_L_ stability via USP40. Additionally, GMEB1 inhibited the activation of pro-caspase 8 and apoptosis in non-small cell lung cancer (NSCLC) cell via CFLAR_L_ stabilization. Also, GMEB1 inhibited the formation of DISC upon TRAIL activation. CFLAR_L_ enhanced the binding of GMEB1 and CASP8. Downregulation of GMEB1 inhibited A549 xenograft tumor growth in vivo.

**Conclusions:**

Our findings show the de-ubiquitinase USP40 regulates the ubiquitination and degradation of CFLAR_L_; and GMEB1 acts as a bridge protein for USP40 and CFLAR_L_. Mechanistically, we found GMEB1 inhibits the activation of CASP8 by modulating ubiquitination and degradation of CFLAR_L_. These findings suggest a novel strategy to induce apoptosis through CFLAR_L_ targeting in human NSCLC cells.

**Electronic supplementary material:**

The online version of this article (10.1186/s13046-019-1182-3) contains supplementary material, which is available to authorized users.

## Background

Glucocorticoid modulatory element-binding protein 1 (GMEB1) was originally identified as a nuclear protein with a molecular weight of 88 kDa [1–3]. GMEB1 interacts with GMEB2 and binds to the promoter sequence glucocorticoid modulatory element (GME) of the tyrosine aminotransferase (TAT) gene to modulate glucocorticoid receptor transactivation [4]. GMEB1 also exists in the cytosol and functions at the protein level. It interacts with the heat shock protein HSP27, but the function is not well studied. GMEB1 also binds to pro-caspases and inhibits their activation and cell apoptosis. However, it is still unknown how GMEB1 does this [5–8].

CFLAR_L_, also known as c-FLIP_L_, plays an important role in extrinsic ligand-induced apoptosis. In this pathway, FasL/tumor necrosis factor-alpha (TNF-α)/TRAIL binds to cell surface receptors, forms Death-Inducing Signaling Complex (DISC) and triggers the caspase-dependent apoptotic pathway. CFLAR_L_ interacts with CASP8 via DED domains and inhibits the activation of CASP8, and thus apoptosis [9–12]. ITCH is an E3 ligase of CFLAR_L_, which enhances apoptosis by targeting CFLAR_L_ through ubiquitin-proteasome pathway [13, 14]. USP8 is a de-ubiquitination enzyme of CFLAR_L_ that enhances stability and inhibits apoptosis induced by extrinsic ligands [15]. Therefore, approaches to promote the ubiquitination and degradation of CFLAR_L_ are potential effective cancer therapies.

In the present study, we showed GMEB1 directly interacted with CFLAR_L_ and increased its stability at the protein level. GMEB1 inhibited the activation of pro-caspase 8 via CFLAR_L_. We found the de-ubiquitination enzyme USP40 bound to CFLAR_L_ and GMEB, which enhanced the interaction between USP40 and CFLAR_L,_ resulting in reduced ubiquitination and degradation of CFLAR_L_. These findings suggest GMEB1 inhibits the activation of CASP8 and apoptosis via CFLAR_L_, which highlights potential implications for lung cancer therapy.

## Methods

### Reagents and antibodies

SAHA was purchased from Sigma (US). CHX, MG132 and E64D were purchased from Selleck (US). PARP (#9542), Caspase 8 (9746 L) antibodies were purchased from Cell Signaling Technology (US). Caspase 3 (NB100–56708) antibody was purchased from Imgenex (US). GMEB1 (sc-376,775), USP40 (sc-514,248) and FLIP_L_ (sc-8346) antibodies were purchased from Santa Cruz (US). CFLAR_L_ (ALX-804-961-0100) antibody was purchased from Enzo (US). HA (D110004) tag antibody was purchased from Sangon Biotech (China). FLAG (F7425) tag antibody was purchased from Sigma (US). His (D291–3) tag antibody was purchased from MBL (Japan). USP8 (27791–1-AP) and FADD (14906–1-AP) antibodies were purchased from proteintech (US).

### Cell lines and cell culture

The human NSCLC cell lines A549, H1299, H1792. H157, H460, Calu-1 and HEK293FT cell lines were originally obtained from the American Type Culture Collection (ATCC). A549 and H1792 cell lines have been authenticated in Microread Gene Technology by STR analysis. The NSCLC cells were grown in monolayer culture in RPMI 1640 with 5% FBS (Gibco, US) at 37 °C in a humidified atmosphere consisting of 5% CO_2_ and 95% air. HEK293FT cells were grown in DMEM with 5% FBS (Gibco, US) at 37 °C.

### RNA interference and plasmid transfection

The GMEB1 siRNA targets the sequence: 5′-GCACCAAAUUUGAUCUUCU-3′ and 5′-GCACACACAUUUGGCCUAA -3′; USP40 siRNA targets the sequence 5′-GCAGCAAAGUCGGCCAAAU-3′ and 5′-GGAUGCAGCUAACAUUGAA-3′. The siRNAs were synthesized by GenePharma and used as the manufacture’s protocol.

GMEB1 and USP40 coding regions were amplified by PCR from A549 genomic DNA using following primers:

GMEB1 sense: CGGATCCGCCGCCACCATGGCTAATGCAGAAGTGAG

GMEB1 antisense: CCTCGAGTTAATCCTCTAAGACCACAATC

USP40 sense: CTAGCTAGCGCCGCCACCATGTCACTTTTTTTAAGGGTAG

USP40 antisense: CGCGGATCCTTATCTGAAGCTCCCCACG

HA tag was cloned to the N-terminal of GMEB1, His tag was cloned to the C-terminal of USP40. His-tagged, FLAG-tagged and GST-tagged CFLAR_L_ were cloned previously by our team.

### Western blot analysis

Cells were harvested and rinsed with pre-chilled PBS on ice. They were lysed in lysis buffer on ice for 30 min and then purified via centrifugation for 15 min at 4 °C. Samples of the whole-cell protein lysates (35 μg) were prepared for SDS-PAGE and transferred to a polyvinylidene fluoride (PVDF) membrane by electro blotting. The proteins were probed with the appropriate primary and secondary antibodies. Antibody binding was detected by an HRP system according to the manufacturer’s protocol [16].

### Immunoprecipitation

Cells were lysed in lysis buffer (20 mM Tris-HCl, pH 7.5; 150 mM NaCl; 1 mM Na_2_EDTA; 1 mM EGTA; 2.5 mM sodium pyrophosphate; 1 mM β-glycerophosphate; 1 mM Na_3_VO_4_; 0.5% Triton) on ice for 30 min then purified via centrifugation for 15 min at 4 °C. The supernatants were incubated with antibody at 4 °C for 1 h. Then the mixture was incubated with protein A beads (ThermoFisher) at 4 °C for 2 h. The beads were washed twice with 1 ml of lysis buffer. 20 μl 2 × SDS buffer were added for elution (100 °C, 10 min). Samples were centrifuged for western blot analysis.

### GST pull-down assay

Cells were lysed in lysis buffer (20 mM Tris-HCl, pH 7.5; 150 mM NaCl; 1 mM Na_2_EDTA; 1 mM EGTA; 2.5 mM sodium pyrophosphate; 1 mM β-glycerophosphate; 1 mM Na_3_VO_4_; 0.5% Triton) on ice for 30 min, then purified via centrifugation for 15 min at 4 °C. The supernatants were incubated with rotation in 20 μl of Glutathione Sepharose beads (GE) at 4 °C for 2 h. Beads were washed twice with 1 ml of lysis buffer. 20 μl 2 × SDS buffer was added to beads for elution (100 °C, 10 min). Samples were centrifuged for western blot analysis.

### Immunofluorescence

Cells were fixed with PHEMO buffer (0.025 M HEPES, 0.068 M PIPES, 0.003 M MgCl_2_·6H_2_O, 0.015 M EGTA·Na_2_, 10% DMSO, pH adjusted to 6.8. Additional reagents were added before use, with a final concentration as follows: 0.05% glutaraldehyde, 0.5% Triton X-100, 3.7% formaldehyde) for 10 min at room temperature before washing with PBS for 3 times. Then, cells were incubated with blocking buffer (3% BSA) for 30 min at room temperature. Afterward, cells were incubated with primary antibodies against CFLAR_L_ (Santa Cruz, dilution at 1:500) for 1 h at room temperature. After washing with PBS for 3 times, cells were incubated with another primary antibodies GMEB1 (Santa Cruz, dilution at 1:500) or USP40 (Santa Cruz, dilution at 1:500) for 1 h. Alex Fluor 488 (Green) and Alex Fluor 568 (Red)-conjugated secondary antibodies were then applied and incubated at room temperature for 1 h. Cell nuclei were stained with DAPI. Images were captured using a confocal microscope (ZEISS, LSM700).

### Flow cytometry analysis

Annexin V-FITC Apoptosis Detection Kit (Biobox Biotech, Nanjing, China) was used for cell apoptosis analysis according to the manufacture’s protocol.

### CASP8 activity detection

A549 cells were prepared for CASP8 activity according CASP8 Activity Apoptosis Assay Kit protocol (Sangon Biotech, Shanghai, China).

### Tumor xenograft model

Fifteen female athymic nu/nu 4-week-old mice were purchased from Vital SPF Biotechnology (Beijing, China). For tumor xenograft establishments, A549 cells overexpressing green fluorescent protein or shGMEB1 RNA (1 × 10^6^ cells/100 μL) were subcutaneously injected into the right side of the abdominal region of mice. Weight of mice and tumor size were detected every 2 days. The tumor volume was calculated as V = π × (length×width^2^)/6.

### Statistical analysis

GraghPad Prism version 5.00 was used for statistical analysis. All data are presented as the mean ± SD. Differences between groups were identified using Student’s t-test. *P* < 0.05 was considered statistically significant.

## Results

### SAHA treatment reduced GMEB1 and CFLAR_L_ protein levels in NSCLC cells

**Fig. 1:**
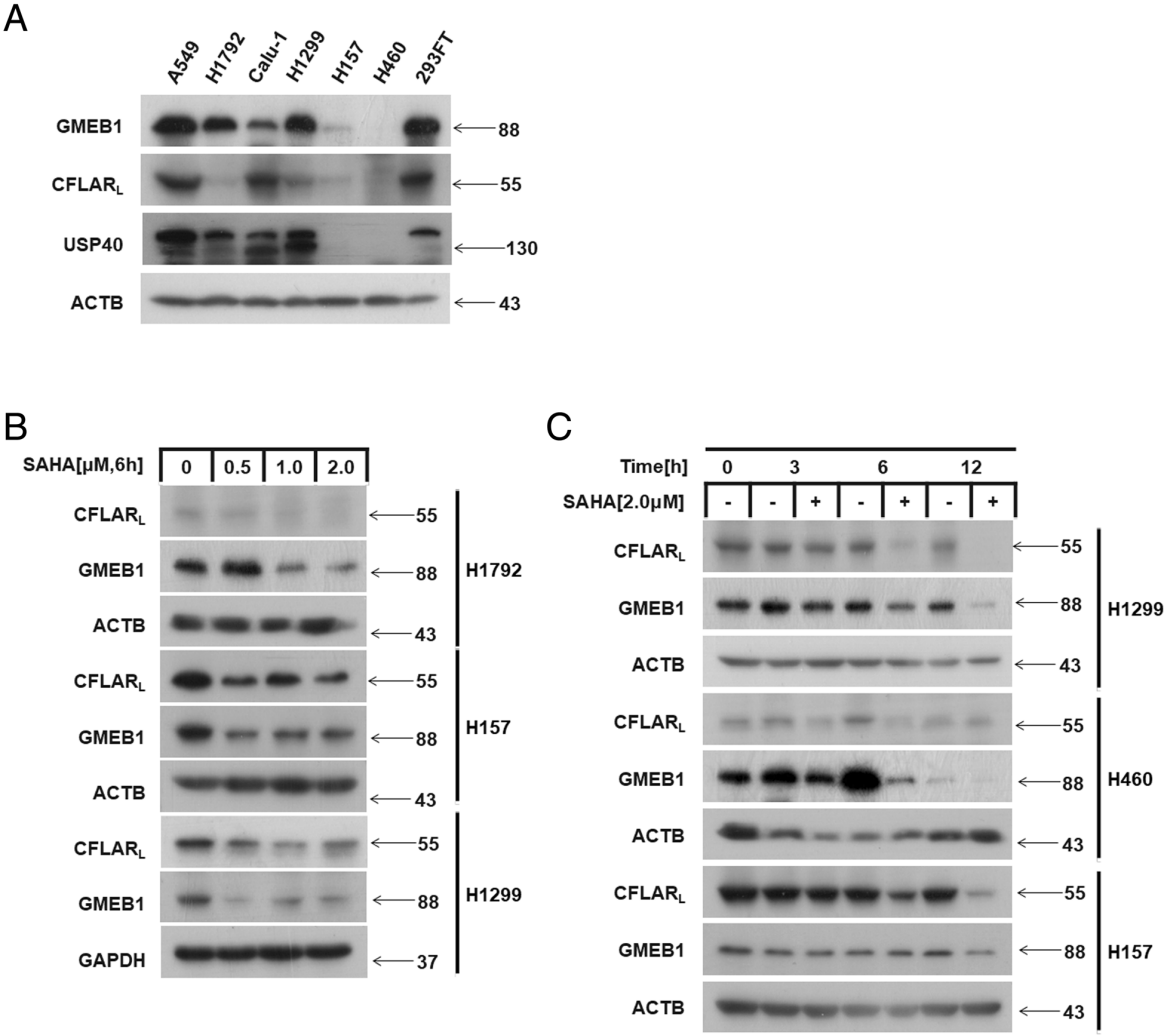
SAHA treatment reduced GMEB1 and CFLAR_L_ protein levels in NSCLC cells. **a** The protein levels of GMEB1, CFLAR_L_ and USP40 were measured in 6 non-small cell lung cell lines and HEK293FT cell line by western blot. GMEB1 and CFLAR_L_ protein levels decreased after SAHA treatment in dose-dependent (**b**) and time-dependent manner (**c**) in different NSCLCs

To examine the interaction between GMEB1 and CFLAR_L_, we measured the protein levels in six NSCLC lines: A549, H1792, Calu-1, H1299, H157, and H460. Western blot assay showed GMEB1 protein levels were high in A549, H1792, Calu-1 and H1299 cells. CFLAR_L_ protein levels were high in A549 and Calu-1 cells (Fig. 1a). CFLAR_L_ protein levels positively correlated with GMEB1 (Additional file 1: Figure S1A). SAHA is an inhibitor of histone deacetylase and enhances TRAIL, which induces apoptosis and decreases CFLAR protein [17–19]. To detect whether SAHA also decreases GMEB1, we treated NSCLC cells with SAHA at different concentrations and time points. The results show SAHA decreased GMEB1 in a dose-dependent (Fig. 1b and Additional file 1: Figure S1D) and time-dependent manner (Fig. 1c and Additional file 1: Figure S1E). This is consistent with the effect of SAHA on CFLAR_L_. These data indicate SAHA treatment affects GMEB1 and CFLAR_L_ similarly in NSCLC cells.

### GMEB1 enhanced the stability of CFLAR_L_

**Fig. 2:**
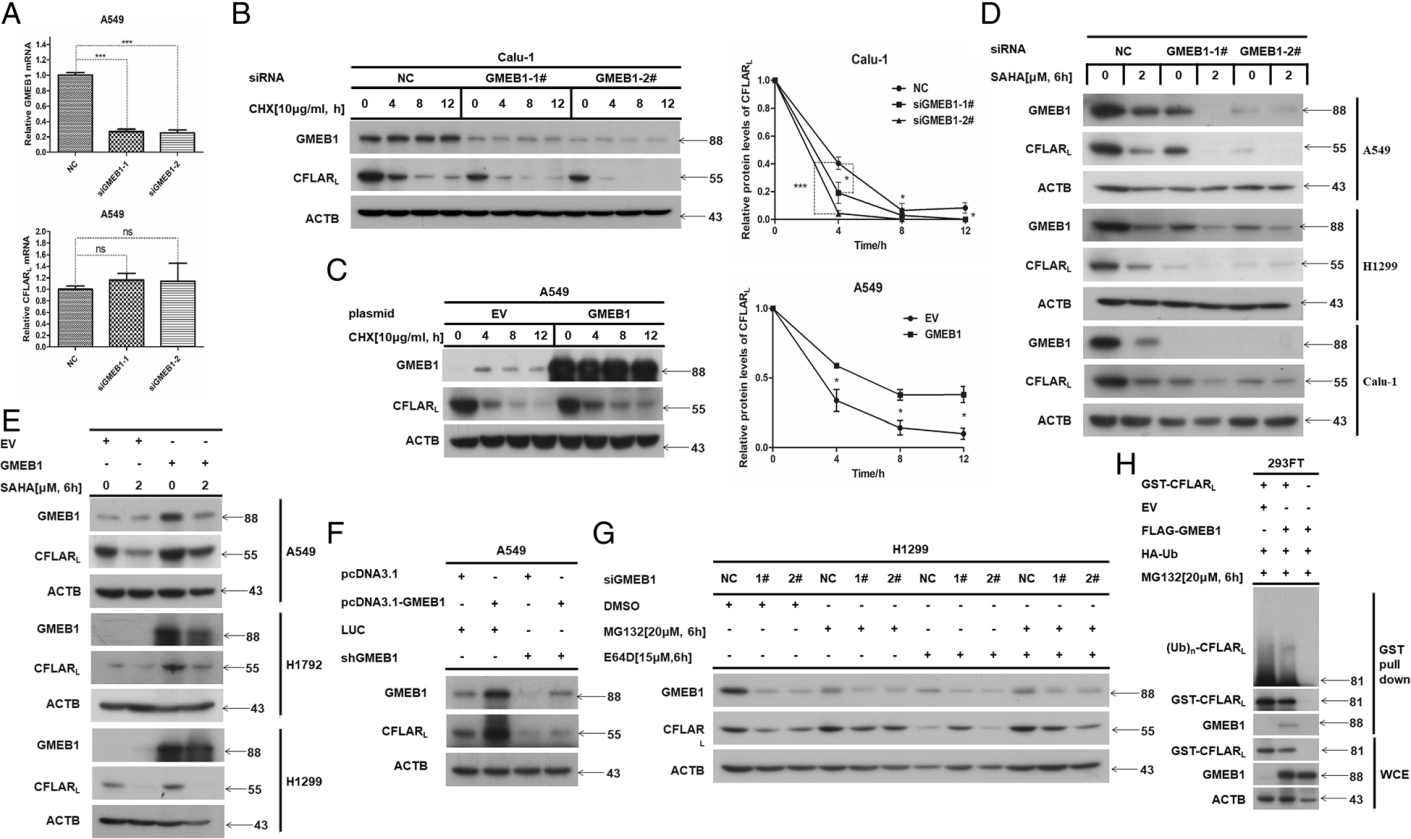
GMEB1 enhanced the stability of CFLAR_L_. **a** Relative mRNA levels of GMEB1 and CFLAR_L_ were determined by quantitative reverse transcription-polymerase chain reaction (q-PCR) in A549 cell line when the cell transfected with GMEB1 siRNA for 24 h. Error bars represent s.d. ***P < 0.001. **b**, **c** Calu-1 and A549 cells were seeded in 6-well plates. GMEB1 siRNA or plasmid were transfected for 24 h and a non-sense siRNA or empty vector was transfected as control. Cells were treated with cycloheximide (CHX) [10 μg/ml] and harvested at different time points (0, 4, 8, 12 h) for western blot analysis. The band intensity of CFLAR_L_ was quantified by Photoshop CS6 and plotted. Experiments were repeated three times, a representative experiment is presented. Every experimental group was compared with the negative control group. Error bars represent s.d. *P < 0.05. **d**, **e** A549, H1299, Calu-1 and H1792 cells were seeded in 6-well plates. GMEB1 siRNA or plasmid were transfected for 24 h. Cells were treated with SAHA [2.0 μM] for 6 h and harvested for western blot analysis. **f** A549-LUC, A549-shGMEB1–1# and A549-shGMEB1–2# were seeded in 6-well plates. GMEB1 plasmid was transfected for 24 h. Cells were harvested for western blot analysis. **g** H1299 cells were seeded in 6-well plates. GMEB1 siRNA was transfected for 24 h. Cells were treated with DMSO, MG132 [20 μM] and E64D [15 μM] for 6 h. Cells were harvested for western blot analysis. **h** HEK293FT cells were prepared for GST pull down assay using GST-CFLAR_L_, FLAG-GMEB1 and HA-Ub plasmids to detect the function of GMEB1 on the ubiquitination of CFLAR_L_

We then characterized the biological function of the interaction between GMEB1 and CFLAR_L_. GMEB1 was originally identified as a transcription factor, so we first determined if GMEB1 regulates CFLAR_L_ at the transcriptional level. GMEB1 and CFLAR_L_ relative mRNA levels were detected using q-PCR in A549 cells; GMEB1 siRNA was transfected for 24 h. Results show that *GMEB1* knock-down did not affect the relative mRNA level of CFLAR_L_ (Fig. 2a). NSCLC cells with *GMEB1* knock-down were treated with CHX [10 μg/ml] for various time points. WB data show *GMEB1* knockdown decreased the stability of CFLAR_L_ (Fig. 2b), while overexpression of GMEB1 increased the stability of CFLAR_L_ (Fig. 2c). This confirms GMEB1 enhances the stability of CFLAR_L_ at post-translational level. Next, we knocked down *GMEB1* by siRNA in A549, H1299 and Calu-1 cell lines and treated cells with SAHA [2.0 μM] for 6 h. Results show *GMEB1* knockdown decreased CFLAR_L_ protein level (Fig. 2d). Overexpression of GMEB1 upregulated CFLAR_L_ protein (Fig. 2e). To confirm the effect of GMEB1 on CFLAR_L_, we knocked down *GMEB1* using GMEB1 shRNA in A549 cell lines and overexpressed GMEB1 using plasmid. We found that GMEB1 overexpression rescued the reduced CFLAR_L_ protein level caused by GMEB1 knockdown (Fig. 2f). These data indicate GMEB1 plays a role in maintaining the protein level of CFLAR_L_.

Most protein degradation occurs in either the ubiquitination-proteasome or ubiquitination-lysosome pathway. As such, de-ubiquitinases inhibit the effective degradation of proteins. Therefore, we focused how GMEB1 affects the ubiquitination of CFLAR_L_. First, we knocked down *GMEB1* in H1299 cells and treated them with DMSO, MG132 [20 μM] and E64D [15 μM] for 6 h. MG132 inhibits the degradation of proteins by blocking proteasomes, and E64D inhibits the degradation of proteins via lysosomes. Western blot analysis shows MG132 treatment rescued the reduced CFLAR_L_ protein level caused by *GMEB1* knockdown. This indicates CFLAR_L_ is degraded through the proteasome pathway when GMEB1 protein levels are decreased (Fig. 2g). In addition, we designed a co-IP assay to determine whether GMEB1 affects the ubiquitination of CFLAR_L_. Data show overexpression of GMEB1 decreased the ubiquitination of CFLAR_L_ (Fig. 2h). Thus, we propose GMEB1 enhances the stability of CFALR_L_ by modulating its ubiquitination level.

### GMEB1 physically interacted with CFLAR_L_ in NSCLC cells

**Fig. 3:**
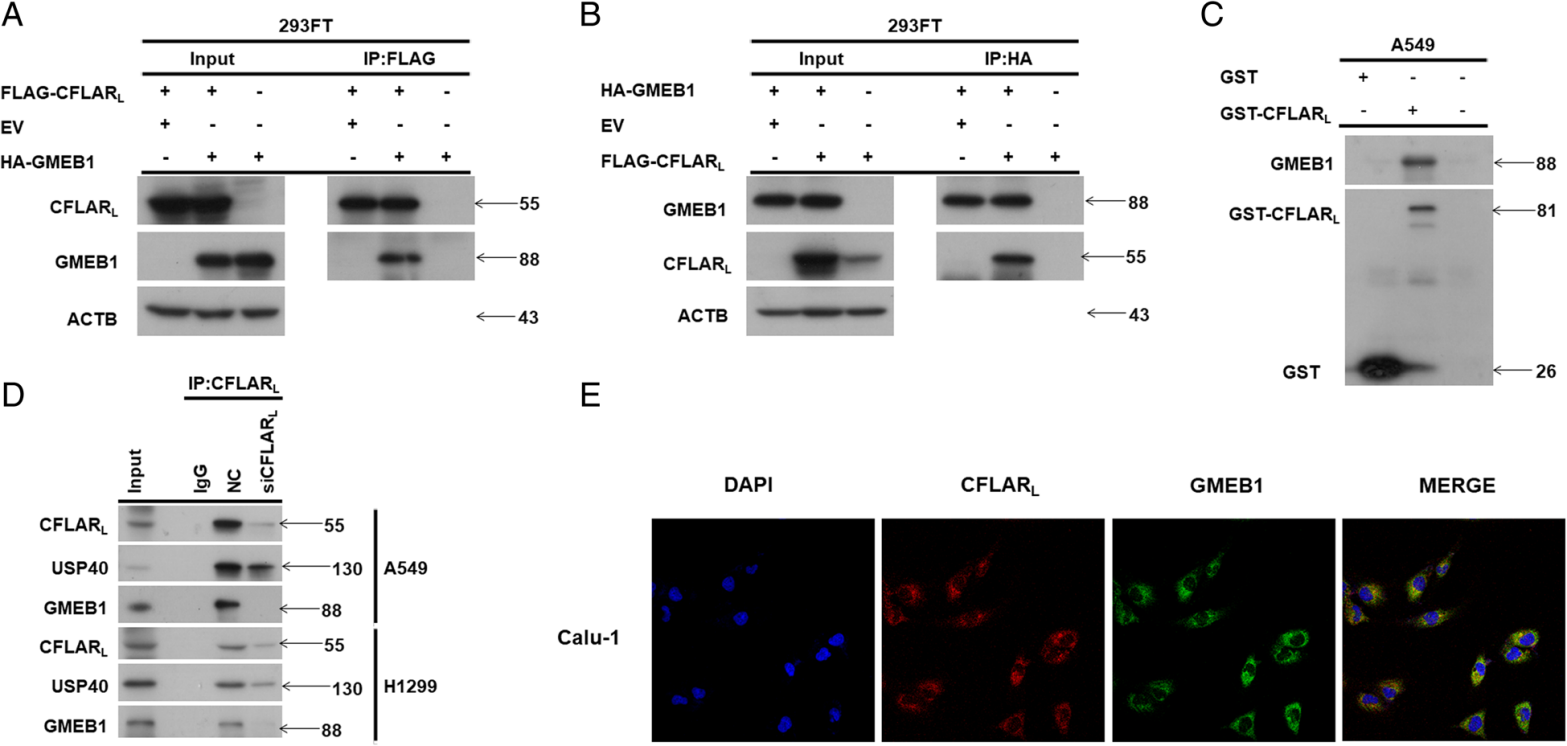
GMEB1 physically interacted with CFLAR_L_ in NSCLC cells. **a**, **b** Co-IP assays were conducted in HEK293FT cells using FLAG-CFLAR_L_ and HA-GMEB1 plasmids. **c** GST-pull down assay was conducted in A549 cells using GST-CFLAR_L_ plasmids. **d** IP assays was conducted in A549 and H1299 cells using anti-FLIP_L_ (Santa Cruz, US) antibody. **e** Calu-1 cells were fixed and subjected to indirect immunofluorescence staining with anti-FLIP_L_ (Santa Cruz, US) and GMEB1 (Santa Cruz, US). The red signal (CFLAR_L_) was obtained with anti-rabbit IgG Alexa 568-conjugated secondary Ab, and the green signals (GMEB1) were obtained with anti-mouse IgG Alexa 488-conjugated secondary Ab. Nuclei were stained with DAPI

GMEB1 interacts with CASP8 and inhibits its activation. *CFLAR*_*L*_ gene has high homology with *CASP8* gene, and the proteins display similar structures that may confer interaction with each other through DED domains. Thus, we determined if GMEB1 and CFLAR_L_ bind one another via a co-immunoprecipitation (co-IP) assay in HEK293FT cells. The data show that HA-tagged GMEB1 interacted with FLAG-tagged CFLAR_L_ (Fig. 3a and b). After GST-tagged CFLAR_L_ was pulled down with Glutathione Sepharose beads, GMEB1 was detected using WB assay, indicating GMEB1 physically interacted with CFLAR_L_ (Fig. 3c). An additional IP assay using A549 and H1299 cells (Fig. 3d) shows that endogenous CFLAR_L_ interacted with endogenous GMEB1. To further evaluate the interaction between GMEB1 and CFLAR_L_, immunofluorescence staining experiments were conducted in Calu-1 cells. Results show GMEB1 localized in the cytosol. GMEB1 and CFLAR_L_ were co-localized in the cytosol (Fig. 3e). We determined which domains of CFLAR_L_ are required for this binding. Our data indicated that DED domains of CFLAR_L_ were not necessary for interaction with GMEB1. However, P20 and P12 fragments of CFLAR_L_ interacted with GMEB1 (Additional file 1: Figure S2A, B and C). Additional results show the N-terminal of GMEB1 was essential for interaction with CFLAR_L_ (Additional file 1: Figure S2D and E). And, the fragment 325–573 of GMEB1, which doesn’t interact with CFLAR_L_, didn’t increase the protein level of CFLAR_L_ in A549 cell lines.

### USP40 interacted with GMEB1 and CFLAR_L_

We next aimed to identify the de-ubiquitination enzyme that targets CFLAR_L_. We examined protein level of several de-ubiquitinases (USP4, USP40, USP7, USP8 and USP22) in six NSCLC cell lines and found USP40 was positively correlated with CFLAR_L_ (Fig. 1a and Additional file 1: Figure S1B) and GMEB1 (Fig. 1a and Additional file 1: Figure S1C). We proposed that USP40 modulated the ubiquitination of CFLAR_L_ via its de-ubiquitinase activity. We then examined the interaction between GMEB1 and USP40 using Co-IP assay and found that tagged GMEB1 directly interacted with tagged USP40, which was confirmed by a reverse experiment (Additional file 1: Figure S3A and B). Given that de-ubiquitination enzymes interact with substrates, we then detected if USP40 directly interacted with CFLAR_L_ using Co-IP assay in 293FT cells and found that tagged USP40 did in fact interact with tagged CFLAR_L_. This was also confirmed by a reverse experiment (Additional file 1: Figure S3C and D). IP assay using anti-FLIP_L_ showed that endogenous CFLAR_L_ interacted with endogenous USP40 in A549 and H1299 cells (Fig. 2d). Immunofluorescence staining results indicate that USP40 also co-localized with CFLAR_L_ in cytosol (Additional file 1: Figure S3E). We next aimed to identify the domains of CFLAR_L_ that were required for binding with USP40. Our data show that DED domains of CFLAR_L_ did not interact with USP40. P20 and P12 fragments of CFLAR_L_ interacted with USP40 (Additional file 1: Figures S2A, S3F and S3G).

### USP40 targets CFLAR_L_ for de-ubiquitination

**Fig. 4:**
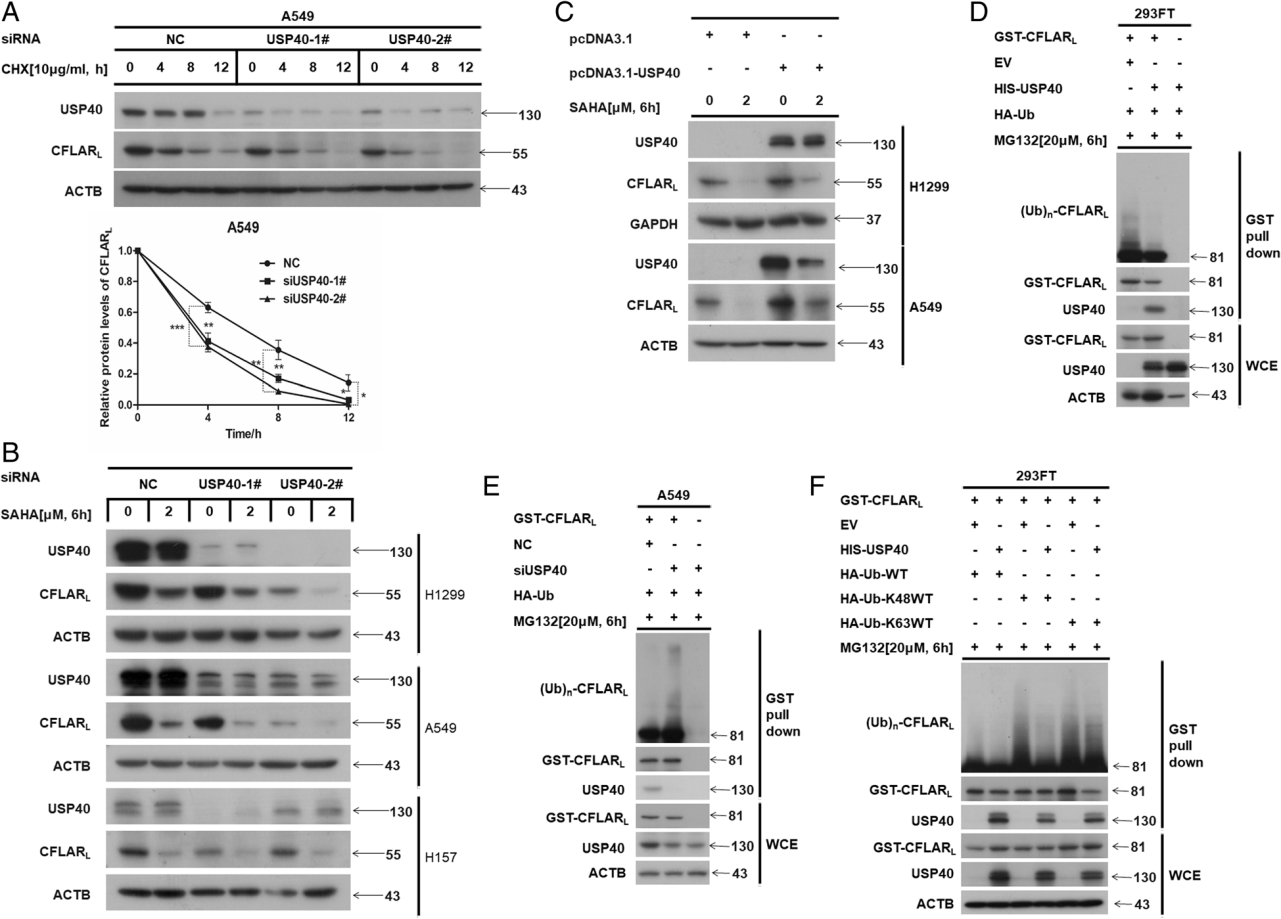
USP40 targeted CFLAR_L_ for de-ubiquitination. **a** A549 cells were seeded in 6-well plates. USP40 siRNA was transfected for 24 h and a non-sense siRNA was transfected as control. Cells were treated with CHX [10 μg/ml] and harvested at different time points (0, 4, 8, 12 h) for western blot analysis. The band intensity of CFLAR_L_ was quantified by Photoshop CS6 and plotted. Experiments were repeated three times, a representative experiment is presented. Every experimental group was compared with the negative control group. Error bars represent s.d. *P < 0.05. **b**, **c** H1299, A549 and H157 cells were seeded in 6-well plates. USP40 siRNA or plasmid were transfected for 24 h. Cells were treated with SAHA [2.0 μM] for 6 h and harvested for western blot analysis. **d**, **e** HEK293FT cells were prepared for GST pull down assays using GST-CFLAR_L_, HIS-USP40 and HA-Ub plasmids or USP40 siRNA to detect the de-ubiquitination of USP40 targeted on CFLAR_L_. **f** HA-Ub-K48WT and HA-Ub-K63WT were cloned from HA-Ub wild type plasmid. Only the 48th or 63rd lysine was natural while all other lysine amino acids were mutated to alanine. GST pull down assay was conducted in HEK293FT cells and the de-ubiquitination model of USP40 targeted on CFLAR_L_ was detected

Previous work showed ITCH targets CFLAR_L_ as an E3 ligase, and USP8 is one of the de-ubiquitination enzymes of CFLAR_L_. Here, we found USP40 was positively correlated with and interacted with CFLAR_L_ in NSCLC cells. Therefore, USP40 may target CFLAR_L_ for de-ubiquitination. To confirm, we first evaluated the function of USP40 on CFLAR_L_ in NSCLC cell lines treated with CHX. Results show that knocking down *USP40* decreased the stability of CFLAR_L_ (Fig. 4a). Furthermore, we knocked down *USP40* in H1299, A549 and H157 cell lines using siRNA and treated cells with SAHA [2.0 μM] for 6 h. Data show protein level of CFLAR_L_ decreased after knockdown of *USP40* (Fig. 4b). Conversely, overexpression of USP40 in H1299 and A549 cell lines increased the protein level of CFLAR_L_ (Fig. 4c). These findings demonstrate USP40 promotes the stability of CFLAR_L_ at the protein level. We used GST pull-down assays to determine the role of USP40 during CFALR_L_ ubiquitination. Results show that overexpression of USP40 decreased the ubiquitination of CFLAR_L_ (Fig. 4d), and knockdown *USP40* increased the ubiquitination of CFLAR_L_ (Fig. 4e). Additional results show that USP40 targeted CFLAR_L_ for K48-linked de-ubiquitination (Fig. 4f). To confirm our proposal, we cloned mutant USP40 (C62A) (Additional file 1: Figure S4A) without enzyme activity [20]. Data show mutant USP40 (C62A) did not increase the protein level of CFLAR_L_ in A549 cell lines (Additional file 1: Figure S4B), but it still interacted with CFLAR_L_ (Additional file 1: Figure S4C). GST pull-down assay results show mutant USP40 (C62A) did not de-ubiquitinate CFLAR_L_ (Additional file 1: Figure S4D). Taken together, these data suggest that USP40 is a de-ubiquitinase of CFLAR_L_.

### GMEB1 promoted the interaction between USP40 and CFLAR_L_

**Fig. 5:**
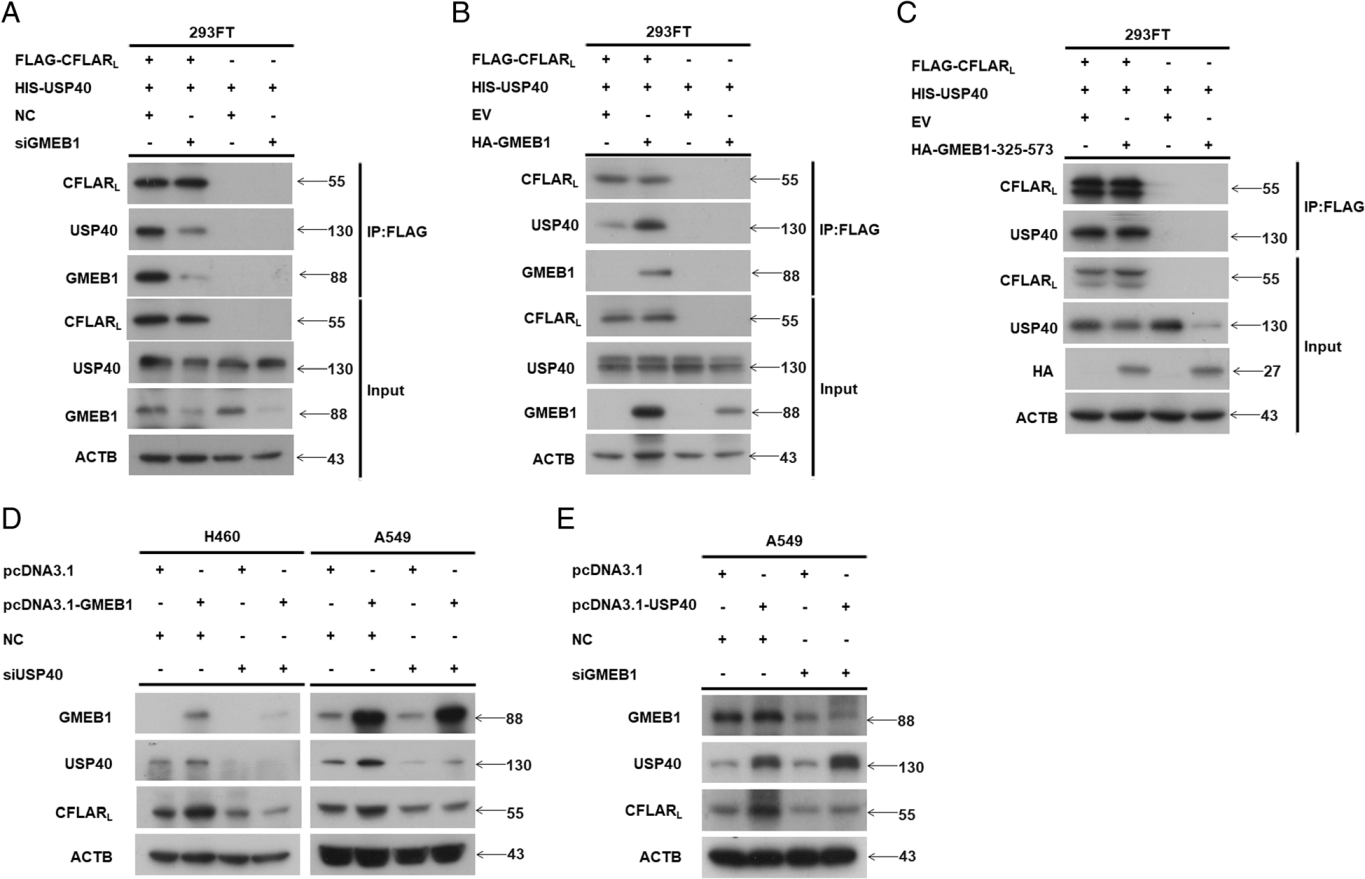
GMEB1 promoted the interaction between USP40 and CFLAR_L_. **a**, **b** GMEB1 siRNA or plasmid were co-transfected with FLAG-CFLAR_L_ and HIS-USP40 in HEK293FT cells. Cells were harvested after 24 h for co-IP assay analysis. **c** HA-GMEB1–325-573 plasmid were co-transfected with FLAG-CFLAR_L_ and HIS-USP40 in HEK293FT cells. Cells were harvested after 24 h for co-IP assay analysis. **d** H460 and A549 cells were seeded in 6-well plates. GMEB1 plasmid and USP40 siRNA were co-transfected for 24 h; non-sense siRNA and pcDNA3.1 were transfected as control. Cells were harvested for western blot analysis. **e** A549 cells were seeded in 6-well plates. USP40 plasmid and GMEB1 siRNA were co-transfected for 24 h; non-sense siRNA and pcDNA3.1 were transfected as control. Cells were harvested for western blot analysis

Our findings show that GMEB1, USP40 and CFLAR_L_ interact to form a complex. We looked into the function of GMEB1 in this complex. Data from co-IP assay in 293FT cells show that knock down of *GMEB1* weakened the interaction between CFLAR_L_ and USP40; overexpression of GMEB1 enhanced the interaction (Fig. 5a and b, respectively). In addition, we found that overexpression of HA-GMEB1 (325–573), which did not interact with CFLAR_L_, had no significant impact on the interaction between CFLAR_L_ and USP40 (Fig. 5c). Our data indicate that GMEB1 acts as an adaptor protein in the complex, and GMEB1 is essential for the interaction between CFLAR_L_ and GMEB1.

Further, we questioned whether GMEB1 affects CFLAR_L_ via USP40. We knocked down *USP40* and overexpressed GMEB1 in H460 and A549 cell lines and found no impact on CFLAR_L_ (Fig. 5d). Using a reverse experiment in A549 cell lines, we showed that USP40 overexpression slightly increased CFLAR_L_ protein when *GMEB1* was knocked down (Fig. 5e). These data indicate GMEB1 confers stability to CFLAR_L_ via USP40.

### GMEB1 inhibited apoptosis via CFLAR_L_ and DISC formation upon TRAIL exposure

**Fig. 6:**
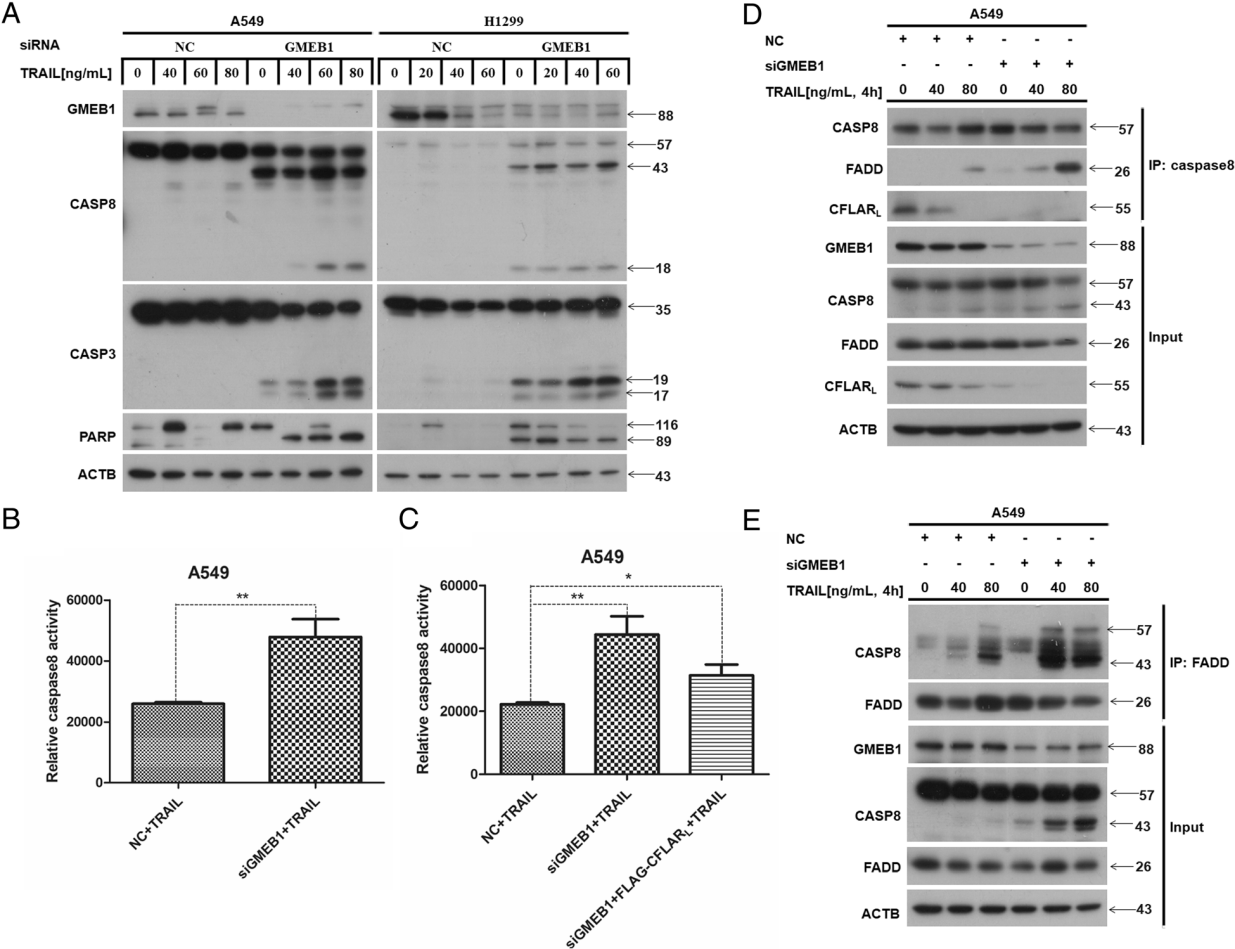
GMEB1 inhibited the formation of DISC upon TRAIL activation. **a** A549 cells and H1299 cells were seeded in 12-well plates. GMEB1 siRNA was transfected for 24 h and a non-sense siRNA was transfected as negative control. Cells were then treated with TRAIL with different concentrations for 6 h and harvested for western blot analysis. **b** A549 cells were seeded in 96-well plates. GMEB1 siRNA was transfected for 48 h and a non-sense siRNA was transfected as negative control. Cells were then treated with TRAIL [40 ng/ml] for 6 h and prepared for CASP8 activity detection. **c** A549 cells were seeded in 6-well plates. GMEB1 siRNA and FLAG-CFLAR_L_ plasmid were co-transfected for 48 h. Cells were then treated with TRAIL [40 ng/ml] for 6 h and prepared for CASP8 activity detection. **d**, **e** A549 cells were seeded in 96-well plates. GMEB1 siRNA was transfected for 48 h and a non-sense siRNA was transfected as negative control. Cells were then treated with TRAIL at different concentrations for 4 h and harvested for IP assay using FADD or CASP8 antibody

GMEB1 inhibits the activation of pro-caspases, but the molecular mechanism is still unclear. Our study demonstrates that GMEB1 interacts with CFLAR_L_ and promotes CFLAR_L_ stability via the de-ubiquitination enzyme USP40. We asked if GMEB1 inhibits apoptosis via CFLAR_L_. GMEB1 siRNA was transfected in A549 and H1299 cell lines for 48 h, and then treated with TRAIL for 6 or 24 h. Western blot results show that *GMEB1* knockdown increased the level of cleaved CASP8, CASP3 and PARP (Fig. 6a). Flow Cytometry analysis shows that *GMEB1* knockdown enhanced apoptosis of A549 induced by TRAIL (Additional file 1: Figure S5B and C). Further, we transfected plasmid FLAG-CFLAR_L_ while knocking down *GMEB1* in A549 cells. Western blot results show that overexpression of CFLAR_L_ decreased protein levels of cleaved CASP8, CASP3 and PARP that were induced by *GMEB1* knockdown and TRAIL treatment (Additional file 1: Figure S5A). Flow Cytometry analysis show that CFLAR_L_ overexpression partially attenuated apoptosis induced by *GMEB1* knockdown, indicating GMEB1 inhibited apoptosis through regulating CFLAR_L_ (Additional file 1: Figure S5D and E). *GMEB1* knockdown increased the activation of CASP8 upon TRAIL treatment in A549 cells (Fig. 6b). And another similar experiment showed that CFLAR_L_ overexpression partially attenuated the activation of CASP8 upon knocking down *GMEB1* and TRAIL treatment (Fig. 6c). We then evaluated the formation of DISC induced by TRAIL treatment. Data show *GMEB1* knockdown promoted the interaction of FADD and CASP8 (Fig. 6d and e).

### CFLAR_L_ is critical for the interaction of GMEB1 and CASP8

**Fig. 7:**
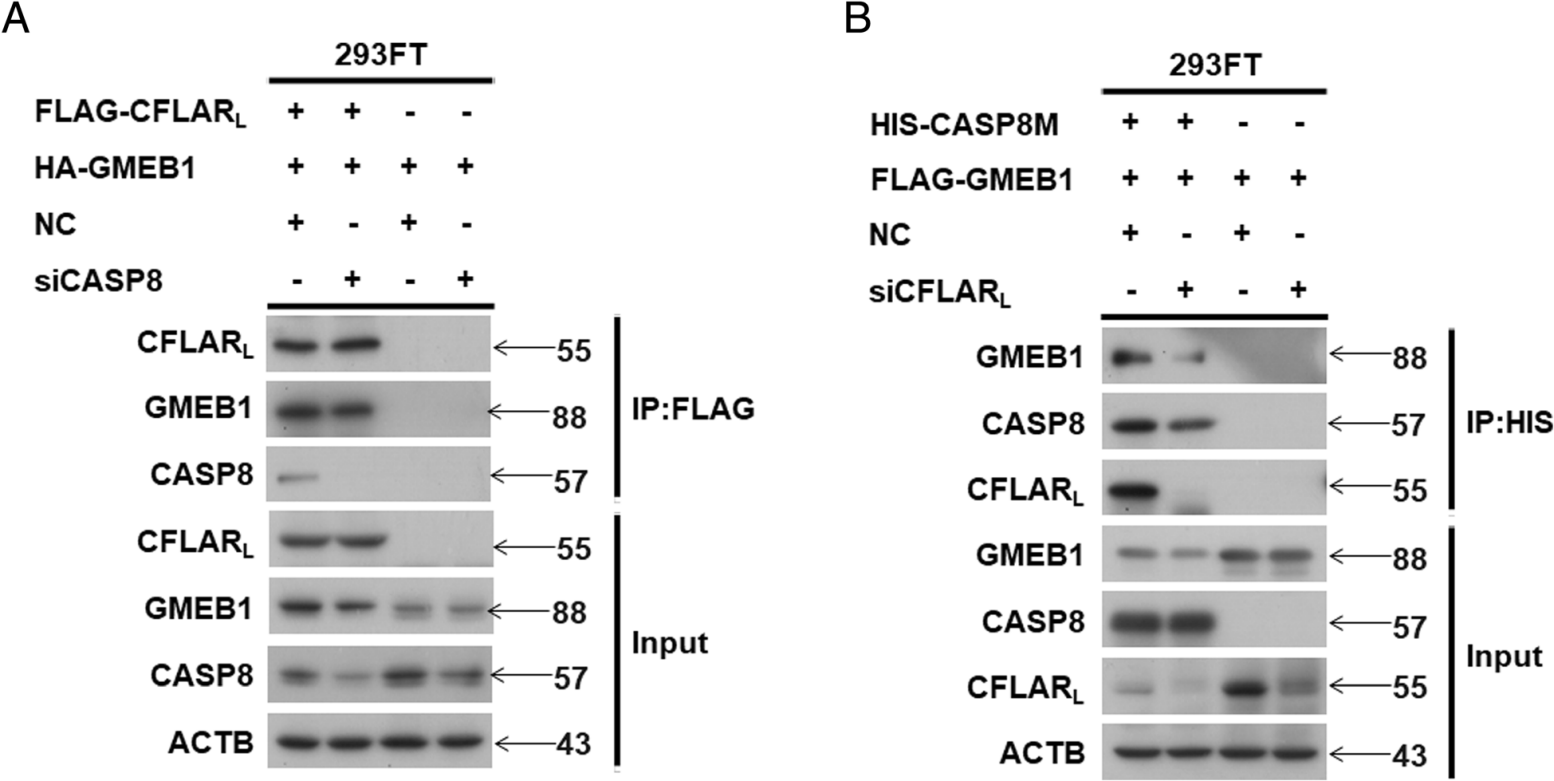
CFLAR_L_ is critical for the interaction of GMEB1 and CASP8. **a** CASP8 siRNA were transfected in HEK293FT cells with FLAG-CFLAR_L_ and HA-GMEB1 plasmids for a co-IP assay. **b** CFLAR_L_ siRNA were transfected in HEK293FT cells with HIS-CASP8M and FLAG-GMEB1 plasmids for a co-IP assay

GMEB1 interacts with pro-caspase 8 and inhibits its activation, but the mechanism is not clear. Our experiments indicated that GMEB1 also interacted with CFLAR_L_, which interacted with pro-caspase 8 and inhibited its activity. We questioned which protein plays the dominant role in the interaction with GMEB1, CASP8 or CFLAR_L_. Co-IP assays were conducted to evaluate the interaction between GMEB1 and CFLAR_L_ while *CASP8* was knocked down using siRNA. Results show no significant change (Fig. 7a). We conducted another co-IP assay to evaluate the interaction between GMEB1 and *CASP8* (using a plasmid HIS-CASP8M that 374 and 384 Aspartic acids mutated to Alanine acids) while *CFLAR*_*L*_ was knocked down using siRNA. Results show *CFLAR*_*L*_ knockdown decreased the interaction between GMEB1 and CASP8 (Fig. 7b). These results confirm CFLAR_L_ directly interacts with GMEB1, and CFLAR_L_ plays a critical role in the interaction of CASP8 and GMEB1.

### Downregulation of GMEB1 inhibited A549 xenograft tumor growth in vivo

**Fig. 8:**
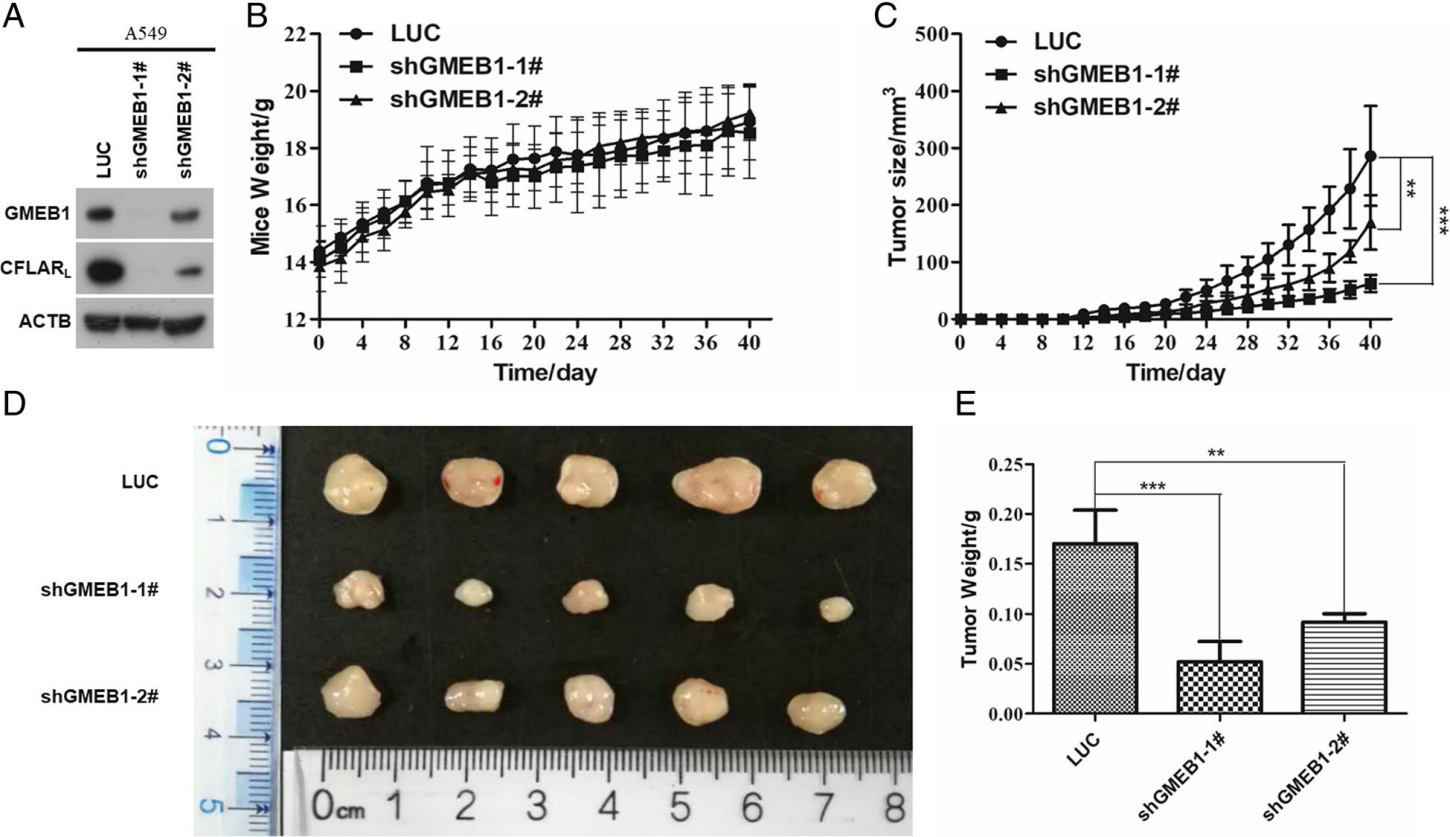
Downregulation of GMEB1 inhibited A549 xenograft tumor growth in vivo. **a** A549 cells stably overexpressing shGMEB1 RNA were seeded in 6-well plates and harvested after 24 h for western blot analysis. **b** A549 cells stably overexpressing shGMEB1 RNA were subcutaneously injected into the right side of the abdominal region of athymic nu/nu mice. Mice weights were measured using electronic balance every 2 days. **c** Tumor sizes were measured with calipers. The error bars represent the SD, ** *P* < 0.01, and *** *P* < 0.001. **d** Tumors were dissected and pictures taken. **e** Tumor weights were measured using electronic balance. The error bars represent the SD, ** *P* < 0.01, and *** *P* < 0.001

To evaluate whether the tumor growth of A549 is regulated by GMEB1 in vivo, A549-LUC, A549-shGMEB1–1# and A549-shGMEB1–2# were subcutaneously injected into the right side of the abdominal region of athymic nu/nu mice. GMEB1 and CFLAR_L_ protein levels were detected in A549 cell lines (Fig. 8a). Mice weights were measured using an electronic balance, results show no significant difference among the three groups (Fig. 8b). Tumor sizes were measured with calipers (Fig. 8c). Results show tumor growth was inhibited after GMEB1 knockdown compared with the control group (Fig. 8d). And, tumor weights also support this finding (Fig. 8e). These results suggest GMEB1 plays a key role in cellular mechanisms related to apoptosis and cancer progress.

## Discussion

GMEB1 is found throughout many cell types with multiple functions that are now being uncovered. GMEB1 and GMEB2 bind to the promoter sequence of the *TAT* gene and modulate GR transactivation. In the cytosol, GMEB1 interacts with HSP27, a protein chaperone with many critical functions in cancer invasion, metastasis, proliferation and apoptosis [21–29]. HSP27 is related to CFLAR_L_ and the activation of caspases [30, 31]. However, the relationship between GMEB1 and HSP27 has not been well studied. Recently, GMEB1 was reported to inhibit cell apoptosis by binding to pro-caspases and inhibiting their activation in cytosol [6]. GMEB1 interacts with pro-caspases via DED domains, but the molecular mechanism is unknown.

CFLAR_L_ is an apoptotic inhibitor protein that interacts with pro-caspase 8 and inhibits its activation; DED domains play important roles in this interaction. The interaction between CFLAR_L_ and pro-caspase 8 inhibits the formation of DISC [15]. As an inhibitor of histone deacetylase, SAHA is effective in treating skin T cell lymphoma clinically. It enhances the acetylation of Ku70 and disrupts the CFLAR/Ku70 complex and then triggers CFLAR poly-ubiquitination and degradation by the proteasome [32, 33]. SAHA treatment in NSCLC cells shows that SAHA decreased CFLAR_L_ and GMEB1 protein levels in a dose-dependent and a time-dependent manner. The results indicate GMEB1 and CFLAR_L_ are positively correlated, which was confirmed by analyzing protein levels in six NSCLC cell lines and a HEK293FT cell line. In addition, GMEB1 protein reduction by SAHA suggests a relationship similar to SAHA and CFLAR_L_.

Given that GMEB1 acts in the nucleus as a transcription factor, we first conducted q-PCR experiments to check the function of GMEB1. We found GMEB1 affected the transcription of CFLAR_L_. We then turned our attention to the cytosol. Knock-down and overexpression experiments showed that GMEB1 promoted the stability and directly regulated the protein level of CFLAR_L_. Our in vitro studies indicated that GMEB1 interacts with CFLAR_L_ outside the nucleus. DED domains of CFLAR_L_ were not necessary for this process; P20 and P12 domains of CFLAR_L_ accounted for the interaction with GMEB1. Our results also show that N-terminal of GMEB1 interacted with CFLAR_L_, and GMEB1 (325–573) fragments, which did not interact with CFLAR_L_, couldn’t increase the protein level of CFLAR_L_. These findings suggest that the function of GMEB1 is dependent on the interaction with CFLAR_L_. Our experiments also indicate GMEB1 affects the degradation of CFLAR_L_ through proteasome pathway. GMEB1 doesn’t have ubiquitin-related enzymatic activity that directly modulates the ubiquitination of CFLAR_L_. This suggests that another enzyme is needed in this process. Therefore, USP40 was emphasized because its expression was positively correlated with GMEB1 and CFLAR_L_ in NSCLC cells.

CFLAR_L_ is an effective target for cancer therapy [9–12]. ITCH, which has important roles in cell immune regulation [34], was identified as an E3 ligase targeting CFLAR_L_. It induces apoptosis by degrading CFLAR_L_ and activating pro-caspase 8. De-ubiquitination is an important protein modification that reverses the ubiquitination of proteins via E1/E2/E3 ligase. De-ubiquitination inhibits the degradation of proteins and drives the fate of substrate proteins [35]. USP8 regulates the morphology of the endosome by ubiquitinating proteins and is also involved in cargo sorting and membrane trafficking at the early endosome stage [36, 37]. USP8 is a de-ubiquitination enzyme of CFLAR_L_ that promotes the stability of CFLAR_L_ and inhibits the activation of pro-caspase 8. Changes in CFLAR_L_ protein levels also affect the formation of DISC and apoptosis induced by extrinsic ligands.

To find the de-ubiquitinase that regulates CFLAR_L_ by GMEB1, we focused on USP8 that was identified as a de-ubiquitinase of CFLAR_L_. But our experiments showed that GMEB1 did not interact with USP8 (Additional file 1: Figure S4E). Western blot data from six NSCLC lines suggested USP40 protein levels are positively correlated with GMEB1 and CFLAR_L_. This underscores the importance of USP40.

Several reports show that USP40 is correlated with late-onset Parkinson’s disease and USP24 [38, 39]. In addition, USP40 affects glomerular permeability in zebrafish [40]. Our results suggest USP40 interacts with both GMEB1 and CFLAR_L_. GMEB1 promoted the binding of USP40 with CFLAR_L_, conferring a role as an adaptor protein. Consequently, USP40 augmented the stability of CFLAR_L_ via its de-ubiquitinase activity. USP40 targeted CFLAR_L_ for K48-linked de-ubiquintion. *USP40* knockdown did not increase the protein level of CFLAR_L_ even though GMEB1 protein level was overexpressed. And, overexpression of USP40 increased the protein level of CFLAR_L_ though *GMEB1* was knocked down. Our data indicate that USP40 is the key protein affecting GMEB1 on CFLAR_L_.

In addition, we also found that *GMEB1* knockdown promoted the activation of pro-caspase 8 and apoptosis induced by TRAIL. CFLAR_L_ attenuated apoptosis that was induced by GMEB1 knockdown, which highlights the function of GMEB1 in inhibiting apoptosis via CFLAR_L_. GMEB1 inhibited the formation of DISC upon TRAIL activation.

Previous studies show GMEB1 interacts with the DED domain of CASP8 and inhibits its activation. Our results show GMEB1 did not interact with DED domain of CFLAR_L_ which has a similar structure with CASP8. However, GMEB1 interacted with CFLAR_L_ via the P20 and P12 domains. Co-IP results showed that *CASP8* knockdown didn’t affect the interaction between GMEB1 and CFLAR_L_, while *CFLAR*_*L*_ knockdown decreased the interaction between GMEB1 and CASP8. These findings suggest that CFLAR_L_ is crucial for the interaction between GMEB1 and CASP8. Thus, GMEB1 interacts with CFLAR_L_, and CFLAR_L_ interacts with CASP8 via DED domains. GMEB1 inhibits the activation of CASP8 via the function of CFLAR_L_.

In vivo data showed *GMEB1* knockdown inhibited the A549 xenograft tumor growth, which also confirmed our results.

## Conclusions

**Fig. 9:**
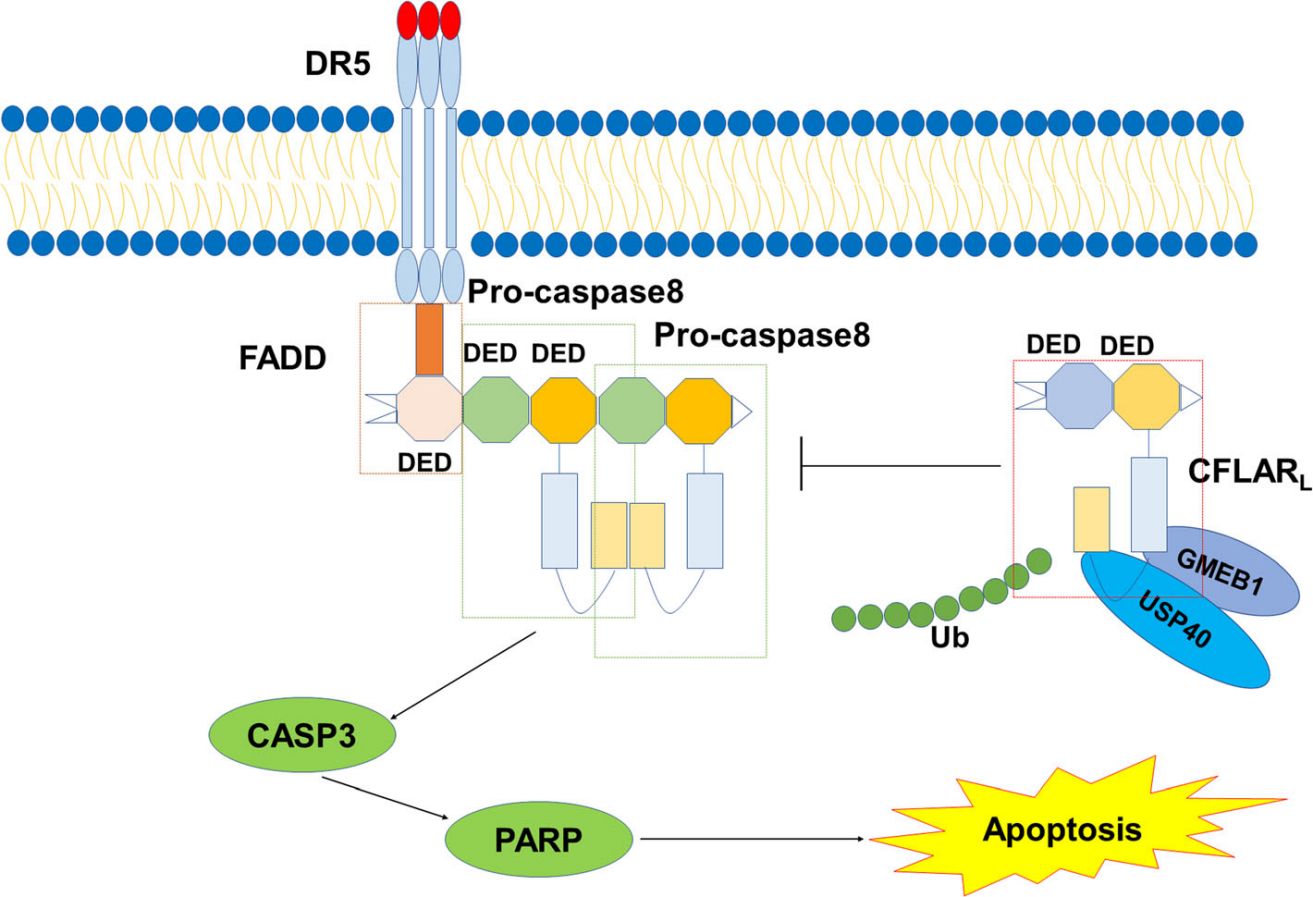
Regulatory mechanism of CFLAR_L_ by GMEB1 and USP40. GMEB1 acts as an adaptor protein to improve the interaction of USP40 and CFLAR_L_. USP40 targets CFLAR_L_ for de-ubiquitination to stabilize CFLAR_L_ protein levels. GMEB1 inhibits the activation of pro-caspase 8 via CFLAR_L_ and inhibits apoptosis

We described the interaction among GMEB1, USP40 and CFLAR_L_ (Fig. 9). We found that GMEB1 promoted the stability of CFLAR_L_ by de-ubiquitinase USP40. USP40 targeted CFLAR_L_ for K48-linked de-ubiquitination. GMEB1 inhibited the activation of CASP8 and apoptosis in NSCLC via CFLAR_L_. CFLAR_L_ promoted the interaction between GMEB1 and CASP8. *GMEB1* knockdown inhibited tumor growth in vivo. These findings provide more in-depth knowledge that serves as potential therapies for cancer.

## Additional file

Additional file 1: **Figure S1.** CFLAR_L_ protein level positively correlated with GMEB1 and USP40 protein levels in NSCLC cell lines. **Figure S2.** CFLAR_L_ directly interact with GMEB1. **Figure S3.** USP40 interacted with GMEB1 and CFLAR_L_. **Figure S4.** USP40 targeted CFLAR_L_ for de-ubiquitination. **Figure S5.** GMEB1 inhibited apoptosis via CFLAR_L_. (DOCX 1283 kb)

## Abbreviations

CFLAR: Cellular FLICE (FADD-like IL-1β-converting enzyme)-inhibitory protein
DISC: Death-inducing signaling complex
HSP27: Heat Shock Protein Family B (Small) Member 1
ITCH: Itchy E3 Ubiquitin Protein Ligase
NSCLC: Non-small cell lung cancer
PARP: Poly ADP-ribose polymerase
