# Supplementary material for: Glucocorticoid modulatory element-binding protein 1 (GMEB1) interacts with the de-ubiquitinase USP40 to stabilize CFLARL and inhibit apoptosis in human non-small cell lung cancer cells
Source: J Exp Clin Cancer Res. 2019 May 2;38:181. doi: 10.1186/s13046-019-1182-3 (PMC6498657; doi:10.1186/s13046-019-1182-3)
Supplement: Supplementary file 1 — Figure S1. CFLARL protein level positively correlated with GMEB1 and USP40 protein levels in NSCLC cell lines. Figure S2. CFLARL directly interact with GMEB1. Figure S3. USP40 interacted with GMEB1 and CFLARL. Figure S4. USP40 targeted CFLARL for de-ubiquitination. Figure S5. GMEB1 inhibited apoptosis via CFLARL. (DOCX 1283 kb) [file 13046_2019_1182_MOESM1_ESM.docx]

**Additional file 1**

**Figure S1: CFLAR_L_ protein level positively correlated with GMEB1 and USP40 protein levels in NSCLC cell lines.** Relative amounts of GMEB1, CFLAR_L_ and USP40 normalized to ACTB in NSCLC cell lines were quantified using Photoshop CS6. The pearson correlation coefficient for the protein expression levels of CFLAR_L_ versus GMEB1 (A) or USP40 (B) and USP40 versus GMEB1 (C) were shown in the picture. (D, E) SAHA treatment was conducted in A549 and Calu-1 cells and cells were then prepared for western analysis.

**Figure S2: CFLAR_L_ directly interact with GMEB1.** The schematic diagram of CFLAR_L_ (A) and GMEB1 (D). (B) FLAG tagged CFLAR_L_ domains and HA-GMEB1 were co-transfected in HEK293FT cells for co-IP assay. (C) CFLAR_L_ domain deletion mutation plasmids were co-transfected with HA-GMEB1 for co-IP assay. (E) FLAG tagged CFLAR_L_ and HA tagged GMEB1 domains were co-transfected in HEK293FT cells for co-IP assay. (F) HA-GMEB1-325-573 and HA-GMEB1-WT plasmids were transfected in A549 cells for western blot analysis.

**Figure S3: USP40 interacted with GMEB1 and CFLAR_L_.** (A, B) HIS-USP40 and HA-GMEB1 were co-transfected in HEK293FT cells co-IP assay analysis. (C, D) HIS-USP40 and FLAG-CFLAR_L_ were co-transfected in HEK293FT cells for co-IP assay analysis. (E) Calu-1 cells were fixed and subjected to indirect immunofluorescence staining with anti-FLIP_L_ (Santa Cruz, US) and USP40 (Santa Cruz, US). The red signal (CFLAR_L_) was obtained with anti-rabbit IgG Alexa 568-conjugated secondary Ab, and the green signals (USP40) were obtained with anti-mouse IgG Alexa 488-conjugated secondary Ab. Nuclei were stained with DAPI. (F) FLAG tagged CFLAR_L_ domains and HIS-USP40 were co-transfected in HEK293FT cells. HIS-USP40 and pcDNA3.1 were co-transfected as control. Cells were harvested for co-IP assay. (G) CFLAR_L_ domain deletion mutation plasmids were co-transfected with HIS-USP40 for co-IP assay.

**Figure S4: USP40 targeted CFLAR_L_ for de-ubiquitination.** (A) The schematic diagram of USP40-C62A. (B) USP40-C62A and USP40-WT were transfected in A549 cells for western blot analysis. (C) FLAG-CFLAR_L_ was co-transfected with HIS-USP40-WT and HIS-USP40-C62A for co-IP assay. (D) GST pull down assay was conducted using GST-CFLAR_L_, HA-Ub, HIS-USP40-WT and HIS-USP40-C62A plasmids in HEK293FT cells. (E) HA-GMEB1 plasmid was transfected in HEK293FT cells for co-IP assay.

**Figure S5: GMEB1 inhibited apoptosis via CFLAR_L_.** (A) A549 cells were seeded in 6-well plates. GMEB1 siRNA and FLAG-CFLAR_L_ were transfected for 48h. Cells were treated with TRAIL for 6h. Cells were harvested for western blot analysis. (B, C) A549 cells were seeded in 6-well plates. GMEB1 siRNA were transfected for 48h. Cells were treated with TRAIL for 24h. Cells were collected for Flow Cytometry analysis. (D, E) A549 cells were seeded in 6-well plates. GMEB1 siRNA and FLAG-CFLAR_L_ plasmid were co-transfected for 48h. Then cells were treated with TRAIL for 24h. Cells were collected for Flow Cytometry analysis.


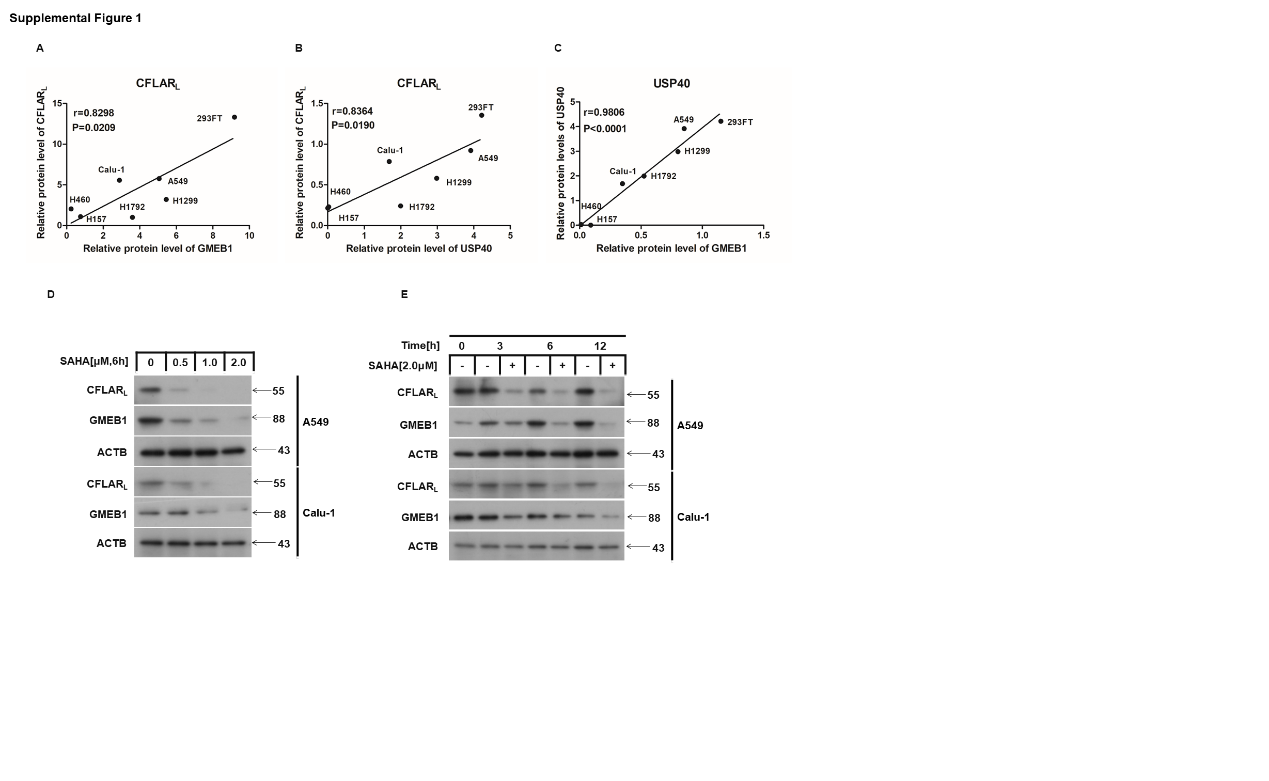
Figure S1


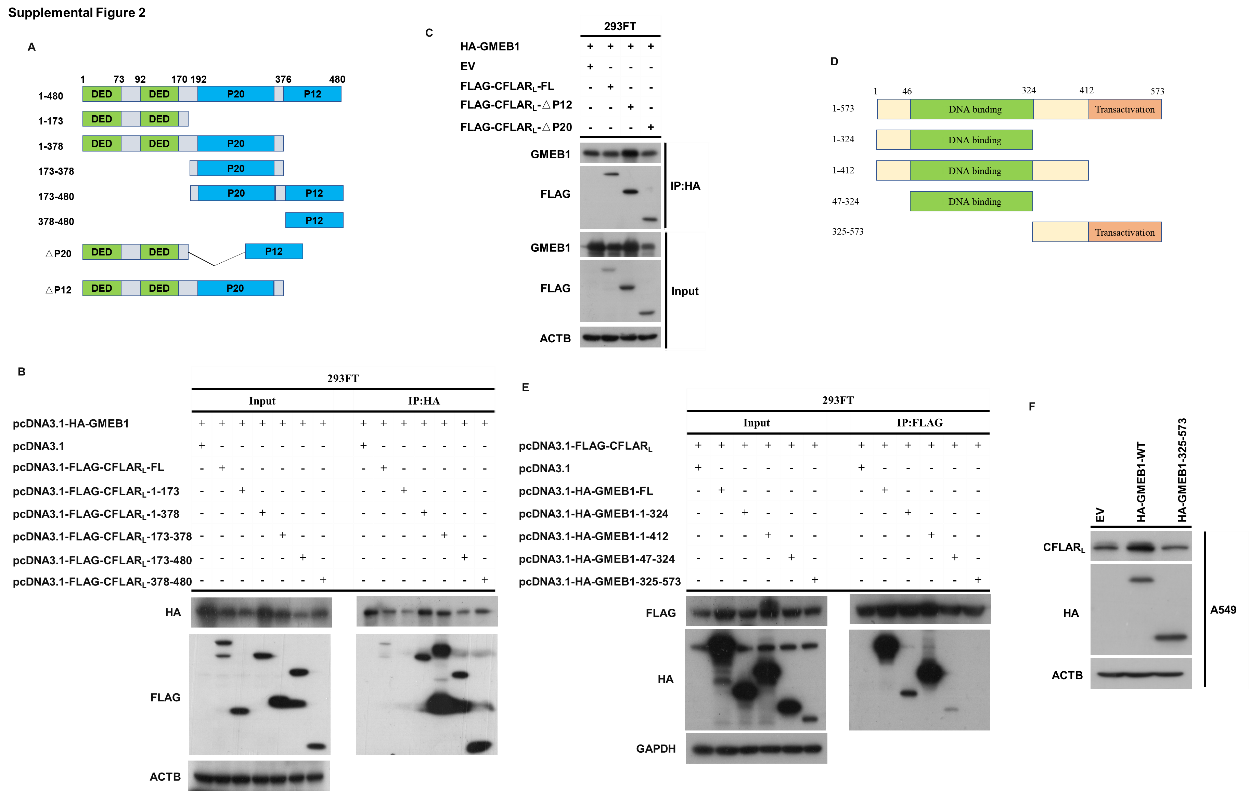


Figure S2


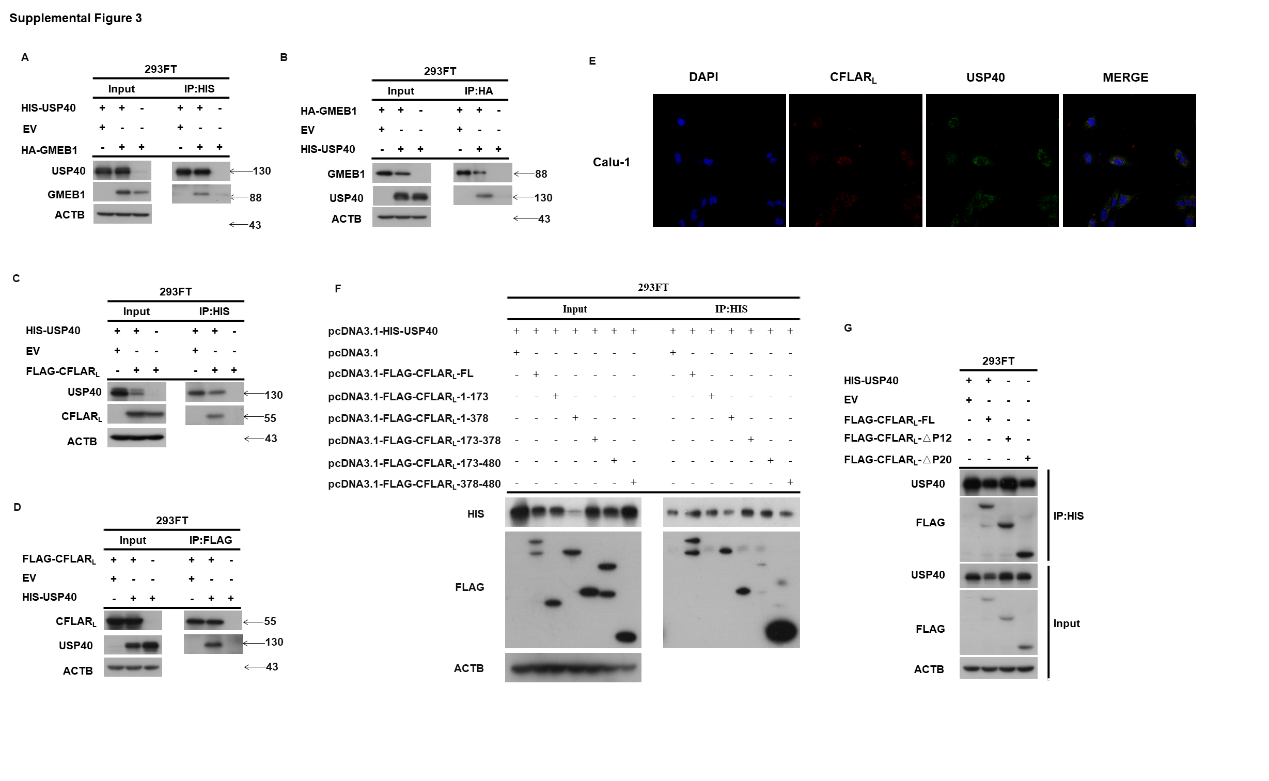


Figure S3


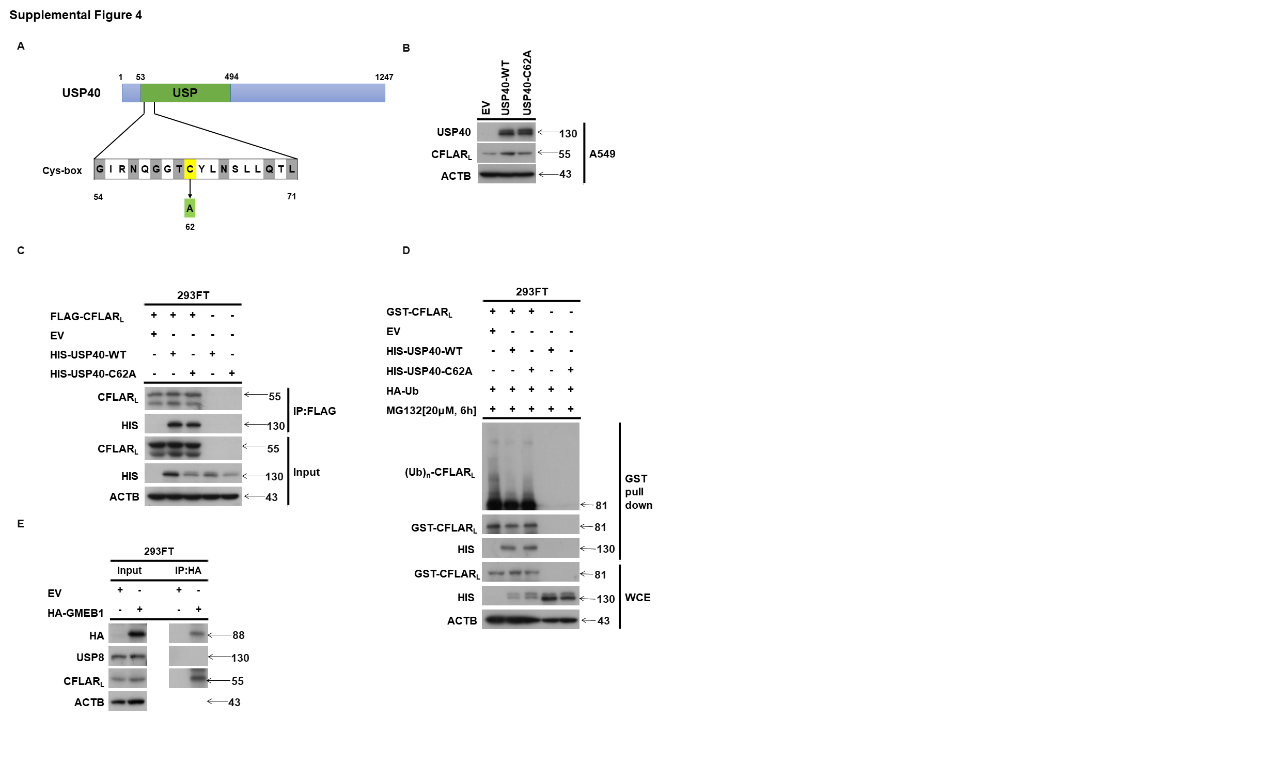


Figure S4


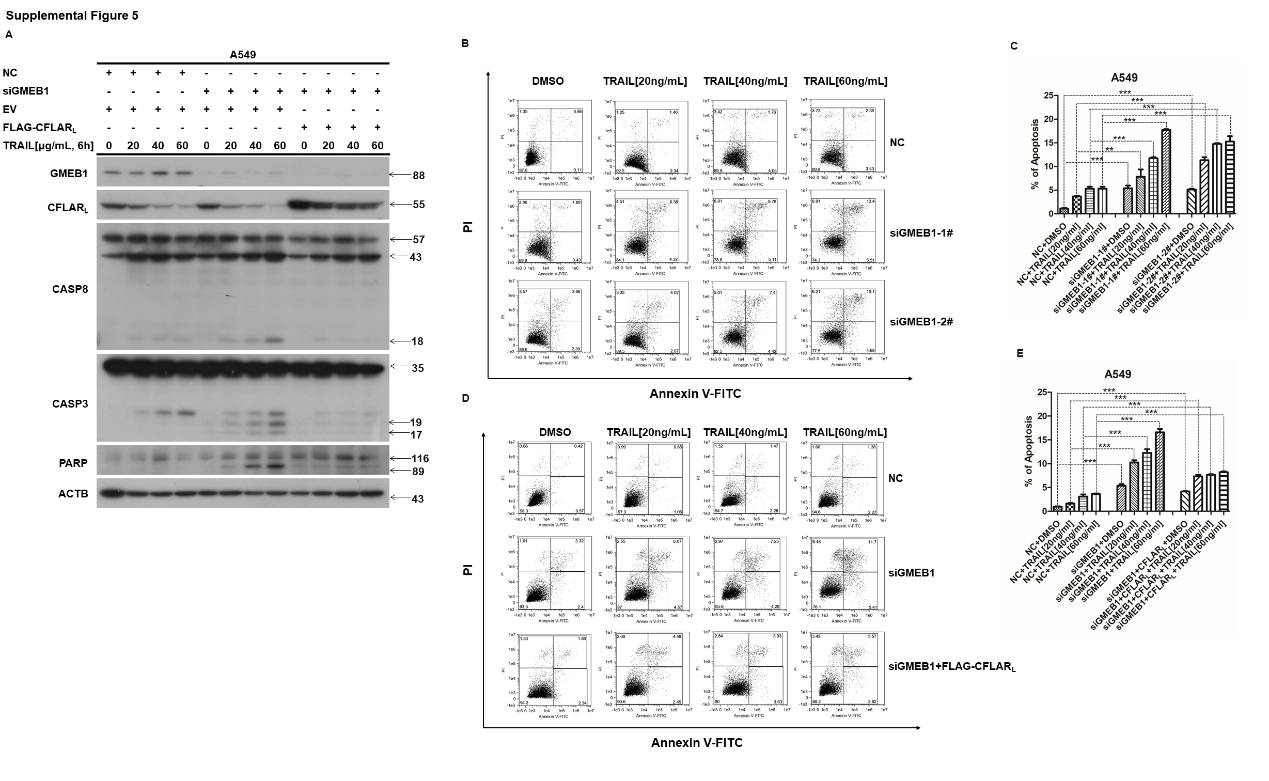


Figure S5
